# Evaluation of five methods to identify composite restorations in human teeth on a forensic purpose—an ex vivo comparative study

**DOI:** 10.1007/s00414-022-02869-z

**Published:** 2022-08-10

**Authors:** Florence C. Auderset, Thomas Connert, Christian Meller, Andreas Filippi, Dorothea C. Dagassan-Berndt

**Affiliations:** 1 Department of Oral Surgery, University Center for Dental Medicine Basel UZB, University of Basel, Basel, Switzerland; 2 Department of Periodontology, Endodontology and Cariology, University Center for Dental Medicine UZB, University of Basel, Basel, Switzerland; 3 Department of Restorative Dentistry, Periodontology, Endodontology and Pediatric Dentistry, School of Dental Medicine, Eberhard-Karls University of Tübingen, Tübingen, Germany; 4 Center for Dental Imaging, University Center for Dental Medicine Basel UZB, University of Basel, Basel, Switzerland

**Keywords:** Forensic odontology, Postmortem dental records, Fluorescence-aided identification technique (FIT), Composite resin materials, Intraoral radiographs

## Abstract

The use of tooth-colored composite resin materials in dentistry is increasing. As composite restorations appear similar to the natural tooth structure, their detection is becoming more challenging. The aim of this study was to compare five diagnostic methods for detection of dental restorations and to create reliable postmortem dental records for forensic purposes. A conventional examination method without a direct light source (CONV), with a dental loupe light (DL), Galilean loupes with a direct light source (GDL), a fluorescence-inducing device (FIT), and intraoral radiographs (RX) were compared. Dentists specializing in conservative dentistry or oral surgery and dentistry students participated. Regarding sensitivity, specificity, positive predictive value, and negative predictive value, FIT and RX showed significantly higher results than CONV, DL, and GDL. RX and FIT showed comparable results in identifying composite restorations but depending on their location. The combination of both methods may lead to even better results. In conclusion fluorescence-inducing devices show good results in identifying composite restorations and therefore should be considered as a standard tool in forensic examinations. Another advantage is their small size, low cost, and mobility. Good illumination and magnification devices are recommended to enhance performance during conventional examinations. Involving dentists specialized in conservative dentistry could contribute to dental identification.

**Supplementary Information:**

The online version contains supplementary material available at 10.1007/s00414-022-02869-z.

## Introduction 

In cases of decayed bodies, where visual recognition or identification by other (fingerprint) means fail, as well as in mass disasters, forensic odontology plays an important role in human identification. Enamel and dentin are recognized the hardest tissues of the body, and therefore, being resistant to decomposition and destruction and often remains as the last structures of the human body [1–3].

The full permanent dentition is comprised of 28–32 teeth, depending on the presence of the wisdom teeth. Each tooth consists of five surfaces (buccal, oral, mesial, distal, occlusal/incisal). Individual presence of the teeth in combination with restorations (fillings of various material, root canal fillings, fixed or removable dentures) result in billions of possible combinations, which show the unique status of teeth in humans.

In cases of odontological identification postmortem records are compared to antemortem records [4]. For many people, an antemortem tooth status is documented by their dentist and has to be stored in most countries for at least 10 years. Individuals with little or no restorative treatment are more challenging to identify than those with numerous and complex treatment. The number of missing teeth is declining as well as the frequency of removable dental prostheses [5–8]. In European countries, the incidence of tooth loss varies demographically (0–2% per year) and tends to diminish [9]. Additionally, or in absence of any dental treatment, anatomical features like tooth shape and size, arrangement, angulation, root angulation, denticles, tooth wear, or bone structure can be defined as individual characteristics and need to be documented in postmortem dental records for correct identification. Dental records include written notes, study casts, dental photographs, dental scans, and radiographs [1–4]. Various surveys in Switzerland from 1992 to 2007 recorded that around 66% of the population went to see their dentist during the last year [5]. Nearly 25% of all people and 56% aged between 15 and 24 years had orthodontic treatment [5]. For patients who had orthodontic or prosthetic treatment, plaster models or scan data should be available and could contribute to dental identification. Comparison of antemortem and postmortem dental records leads to dental identification. Even though there might be some discrepancies between date of the last dental documentation and the date of the death, a postmortem dental documentation enables a focused search of antemortem records. False documentation of dental restorations or false records can make identification impossible [1–4, 10, 11]. In times of DNA technology, teeth are still an important basis of identification. Teeth are quite resistant to environmental factors and provide a fast identification process as well as low costs. For both methods, DNA and teeth, a suspicion of identity has to exist. If no antemortem dental records are available or discrepancies cannot be explained, DNA profiling should be considered [1–3].

As the use of amalgam is disappearing and being replaced with tooth-colored composite resin materials, the recognizability of restorations becomes more challenging [12]. Composite resin materials are available in different shades, transparencies, and colors and fit the natural tooth structure better than ever before. Especially in forensic examinations and caries screenings where time and equipment are often limited, it is important to find a fast and reliable way to correctly diagnose aesthetical perfect composite restorations [13]. Different international classification of teeth and difficulties diagnosing tooth-colored restorations are common potential sources of errors in forensic examinations [1]. Drying of teeth and good illumination are often not enough to identify composite restorations due to the high-quality aesthetics. False-positive or false-negative diagnoses are not unusual, even if dentists work with magnification devices. Galilean loupes with a 2.5 × magnification factor are mostly chosen in dentistry to improve visual acuity [14–16]. Although some experts and the literature praise the benefits of optical magnification, there is very little scientific evidence on this matter [17].

Composite resin materials are composed of inorganic filler particles surrounded by an organic resin matrix. To ensure high aesthetics under all light conditions, rare-earth oxides, well known as fluorescent additives in glass fillers, are added to imitate the physical luminescence properties of the teeth [10, 18]. These fluorescent additives lead to better aesthetic results, but also contribute to better recognition in order to identify or remove composite restorations [19]. Around 80% of commercial composite resin materials display a greater maximum fluorescence property as compared to the natural fluorescence property of enamel or dentin [20]. The best detection wavelength (maximum excitation) of available composite shades of several different brands was observed at 398 ± 5 nm [21]. Using blue-violet light–induced fluorescence during composite removal can prevent overpreparation, contribute to less invasive treatment, and be useful removing composite-bonded orthodontic appliances or trauma splints [19, 22, 23]. Although it is still unusual to use fluorescence-inducing devices for diagnostic purposes, previous studies showed a significant higher detection rate of composite restorations with a fluorescence-aided identification technique (FIT) then with a conventional illumination method [15, 24].

Comparison of ante- and postmortem dental radiographs is a common method in forensic odontology [3].

In Switzerland and other European countries, about half of medical-indicated radiographs are taken by dentists.

This forms a broad basis for dental radiographic documentation of patients [25]. Most composite resin materials show a higher radiopacity, because of added filler particles, than enamel or dentin, which is essential to observe the confine between the composite restoration and the sound tooth structures [12, 26]. Periapical radiographs are usually suffice for postmortem documentation. For ideal comparison, the acquisition of postmortem radiographs should be performed with the same angulation of available antemortem radiographs [4, 27, 28]. Some authors claim that radiographs of tooth-colored restorations may not be enough for dental identification [1], which implicates an additional diagnostic, respectively detecting tool for these kind of restorations.

The aim of this study was to compare five diagnostic methods to identify composite restorations in human teeth on a forensic purpose. Furthermore, the influence of the working field of different dentists and dentistry students was investigated. The null hypothesis is that there is no difference between the methods and the groups.

## Materials and methods

### Tooth models

The tooth models used were the same as described in previous studies of Meller et al. [15] and Leontiev et al.[24]. Thirty-two extracted teeth were mounted on a mandibular and maxillary arch in their anatomical position. A total of 23 composite restorations was placed in 16 of the 32 teeth. A detailed description of used materials and shade are listed in Table 1. For all restorations, OptiBond™ FL (Kerr, Scafati, Italy) was used as bonding agent.

**Table 1:** List of placed composite restorations for the studies of Meller et al. [15] and Leontiev et al. [24]

Localization of the restoration	Black classification	Brand name	Manufacturer	Shade code	Batch number
17 (b)	V	Amaris®	VOCO GmbH	O2	0,845,224
16 (o)	I	Tetric EvoCeram®	Ivoclar Vivadent AG	A2	T26729
15 od	II	Amaris®	VOCO GmbH	O2	0,845,224
12 dp	III	ENAMEL Plus HRI®	GDF—Gesellschaft für Dentale Forschung und Innovationen GmBH	UE2 UD2 UD3	2,014,004,972 2,011,002,646 2,011,001,293
12mbip	IV	IPS Empress Direct®	Ivoclar Vivadent AG	A3D A2E OpalTrans	M14199 M26421 M12328
11 mp	III	Tetric Evo Ceram®	Ivoclar Vivadent AG	A2	T26729
21 bp	V	Tetric Evo Ceram®	Ivoclar Vivadent AG	A2	T26729
21p	I	Tetric Evo Ceram®	Ivoclar Vivadent AG	A2	T26729
22mc	III	Ceram.x Spectra ST-HV	Dentsply Sirona Deutschland GmbH	A2	60701572 N
22 dbip	IV	ENAMEL Plus HRI®	GDF – Gesellschaft für Dentale Forschung und Innovationen GmBH	UE2 UD2 UD3 OBN	2,014,004,972 2,011,002,646 2,011,001,293 2,009,005,029
23 b (V)	V	Tetric Evo Ceram®	Ivoclar Vivadent AG	A3.5D A3 A2	T29541 T11009 T26729
27 ob	I	Venus® Diamond	Heraeus Kulzer GmbH	A2 B3	010,025 010,021
34dc	V	ENAMEL Plus HRI®	GDF—Gesellschaft für Dentale Forschung und Innovationen GmBH	UE2	2,014,004,972
38 b (V)	V	Tetric Evo Ceram®	Ivoclar Vivadent AG	A2 A3.5D	T26729 T29541
37 o	I	Miris® 2	Coltène-Whaledent AG	DS4 ENT	0,145,005 0,140,145
37 b (V)	V	Tetric Evo Ceram®	Ivoclar Vivadent AG	A1	S31518
36 mob	II	ENAMEL Plus HRI®	GDF—Gesellschaft für Dentale Forschung und Innovationen GmBH	UE2	2,014,004,972
36 l	I	Tetric Evo Ceram®	Ivoclar Vivadent AG	A2	T26729
35od	II	ENAMEL Plus HRI®	GDF – Gesellschaft für Dentale Forschung und Innovationen GmBH	UD4 UE2	2,009,004,761 2,014,004,972
35 b (V)	V	IPS Empress Direct®	Ivoclar Vivadent AG	A2E	M26421
34 b (V)	V	IPS Empress Direct®	Ivoclar Vivadent AG	A3D A2E	T29541 M26421
31 dibl	IV	Tetric Evo Ceram®	Ivoclar Vivadent AG	A2 A2D	T26729 P03092
47 o	I	Tetric Evo Ceram®	Ivoclar Vivadent AG	A1 A3	S31518 T11009

**Fig. 1:**
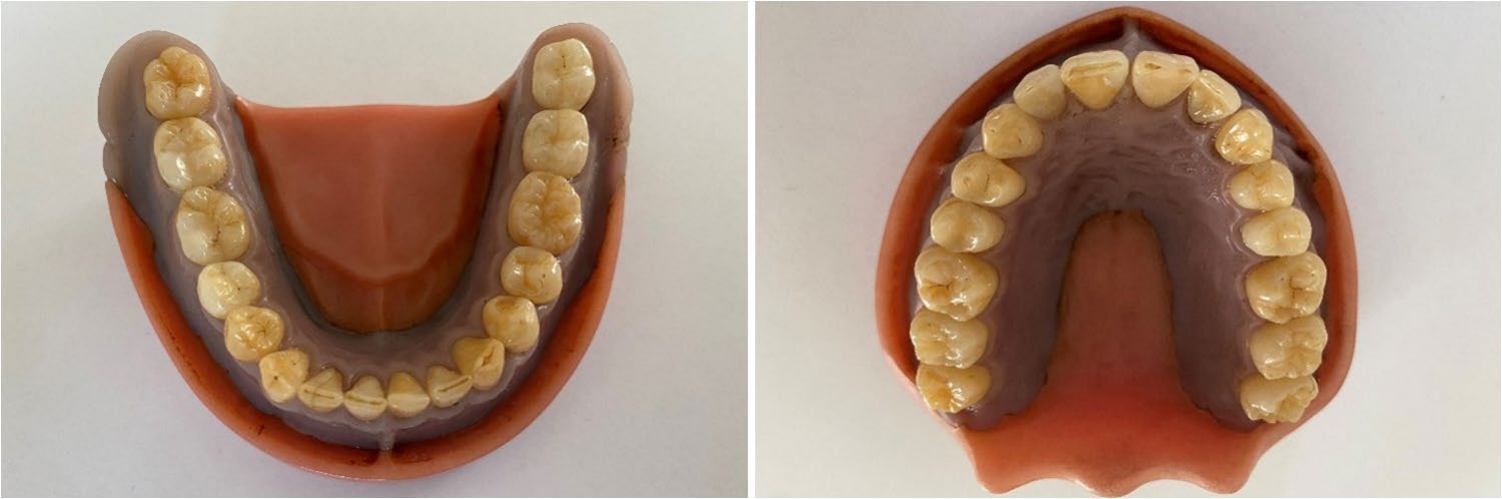
Mandibular and maxillary tooth model

Since the fabrication, the models have been stored in 0.9% saline solution, so there were no changes in tooth color due to dehydration. Regarding margin quality and surface texture, all composite restorations were rated as sufficient. For the use of extracted teeth, human ethics were approved (protocol number EKNZ UBE-15/111). The maxillary and mandibular model are shown in Fig. 1.

As both 46 and 47 had a fracture of the mesiobuccal cusp, new composite restorations have been placed for the present study. The cavity was optimized with a diamond bur in a high-speed handpiece using water cooling, and the composite restorations were placed according to the original protocol with OptiBond™ FL (Kerr, Scafati, Italy) as bonding agent. In this study newly placed composite restorations are presented in Table 2. This resulted in a total of 25 composite restorations in 17 of 32 teeth.

**Table 2:** List of newly placed composite restorations for the present study

Localization of the restoration	Black classification	Brand name	Manufacturer	Shade code	Batch number
46ob	I	Tetric Evo Ceram®	Ivoclar Vivadent AG	A3 A3.5D	T11009 T29541
47ob	I	Tetric Evo Ceram®	Ivoclar Vivadent AG	A2	T26729

Intraoral radiographs of the crowns in both models were taken using an intraoral x-ray system (Heliodent Plus Intraoralstrahler, Dentsply Sirona GmbH, Bensheim, Germany), projected on and developed with a digital intraoral imaging plate system (DIGORA™ Optime DXR-50, SOREDEX, Schutterwald, Germany). Voltage and amperage were set at 70 kV and 7 mA and the exposure time at 0.125 s. Image plate size 0, 1, and 2 were used depending on location (*size 0* = 22 × 31 mm, 628 × 885 pixels, *size 1* = 24 × 40 mm, 685 × 1143 pixels, *size 2* = 31 × 41 mm, 886 × 1171 pixels).

**Fig. 2:**
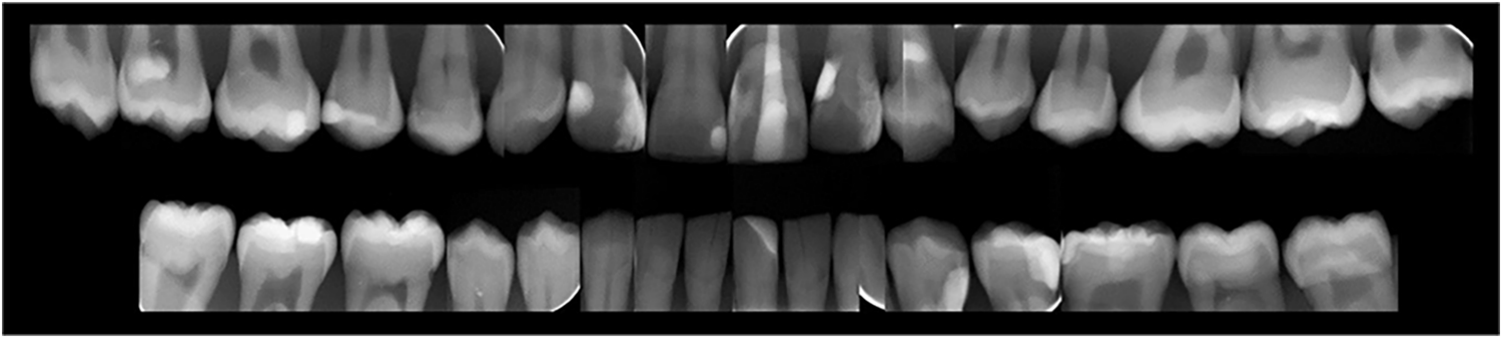
Intraoral radiography status of the crowns of the tooth model

In Fig. 2, the intraoral radiograph’s status is shown. The different composite resin materials appeared all radiopaque on the radiograph expect for the one layer placed on tooth 12.

### Examination procedure

Fifteen examiners composed of five dentists specializing in conservative dentistry (CONS), five dentists specializing in oral surgery (SURG), and as five students (STUD) in their final semester of dentistry participated. All examiners were younger than 35 years and had between 1 and 7 years of professional experience in dentistry. Color blindness and color weakness were assessed by an Ishihara test and led to exclusion. Examiners with visual deficiencies who needed glasses or contact lenses for correction had to wear them during all examinations.

Before the examination, standardized instructions were given to all examiners. The examiners were instructed to record the dental findings by drawing the extent of the diagnosed composite restorations on a dental record. To simulate clinical conditions, the models were fixed in a dental mannequin, kept wet until the examination and rewetted every 60 s during the examination with distilled water to prevent dehydration and changes in color. All examinations were supervised by the same operator to ensure that the procedures were performed according to the study protocol.

The use of four basic instruments was allowed for all methods. The examinations were performed in a room illuminated by ceiling lighting and daylight (500–800 Lux) without direct solar irradiation. An overview of the methods, the used instruments, and the light sources is given in Table 3.

**Table 3:** Overview of the methods and used instruments

	Method	Instruments
1	Conventional examination method (CONV)	Basic instruments -Dental mirror (Mundspiegel TopVision 4P/SS Rhodium, E. HAHNENKRATT GmbH, Königsbach-Stein, Germany) -Sharp Cowhorn Explorer (Deppeler Sonde S 23H, Deppeler SA, Rolle, Switzerland) -Multifunctional syringe for blow-drying -Cellulose wipes
2	Dental loupe light method (DL)	Basic instruments -Safety goggles (Uvex ultra spec, UVEX Arbeitsschutz AG, Basel, Switzerland) -Dental loupe light (Diobright lll, JADENT Dentalvertrieb GmbH, Aalen, Germany), 80′000 Lux
3	Galilean loupe system and dental loupe light method (GDL)	Basic instruments -Galilean loupes, magnification of 2.5x, adjustable eye distance (Keeler Surgical Loupes, Keeler, Malvern, Pennsylvania, USA) -Dental loupe light (Diobright lll, JADENT Dentalvertrieb GmbH, Aalen, Germany), 80′000 Lux
4	Fluorescence-aided Identification Technique (FIT)	Basic instruments -Fluorescence-inducing device λ = 405 nm (SIROInspect, Dentsply Sirona, York, Pennsylvania, USA) -Yellow tinted eyeglasses
5	Intraoral radiography status method (RX)	Basic instruments -X-ray status

The examinations were performed per method with a break of 1 week in between and twice in the same session to calculate the repeatability. This resulted in a total of 150 dental charts (15 examiners × 5 methods × 2 repetitions). The five methods had to be completed in the following sequence: 1 CONV, 2 DL, 3 GDL, 4 FIT, 5 RX. The examiners had no access to their previously completed dental records. Each record was evaluated independently. In between the sessions, the examiners were not allowed to share any information about the study and were not informed that the models had been used for previous studies.

For the GDL, a Galilean loupe system with a 2.5 × magnification factor and an adjustable eye distance mounted as a headband was chosen. The dental loupe light was fixed on the headband for the GDL and for the DL on safety goggles. This enabled wearing of individual eyeglasses during all examinations. The intraoral radiographs status of the tooth models was shown to the examiners during RX and compared with the tooth models. As the radiographs have been taken digitally, they were projected on a screen (HP E243 Monitor, 23.80″, 1920 × 1080 pixels, Hewlett-Packard Company, California, USA). Monitors were calibrated with DenvisQS Software (Version 2.0.2.36, CoSi dental GmbH, Sigmaringen, Germany) to ensure sufficient diagnostic quality.

### Statistical analysis

Statistical analysis was performed using SPSS v.27.0 (IBMCorp., Armonk, New York, USA).

For each method the sensitivity, specificity, positive predictive value (PPV), negative predictive value (NPV), and 95% confidence interval (CI) were calculated based on a filling prevalence of 42%.

PPV and NPV dependent on prevalence (0–100%) were calculated by Bayes’ theorem, based on the sensitivity and specificity values.

To assess the influence of the group (CONS, SURG, STUD) and method (CONV, DL, GDL, FIT, RX) on the sensitivity, specificity, PPV, and NPV, an ANOVA (analyses of variance) model for repeated measurements was applied followed by Bonferroni adjusted post hoc analysis.

The level of significance was set at α = 0.05. Nonparametric 95% confidence intervals (CI) were determined by descriptive statistical analysis.

The interrater reliability was calculated using Fleiss’ Kappa and the intrarater reliability using Cohens’ kappa.

## Results

**Fig. 3:**
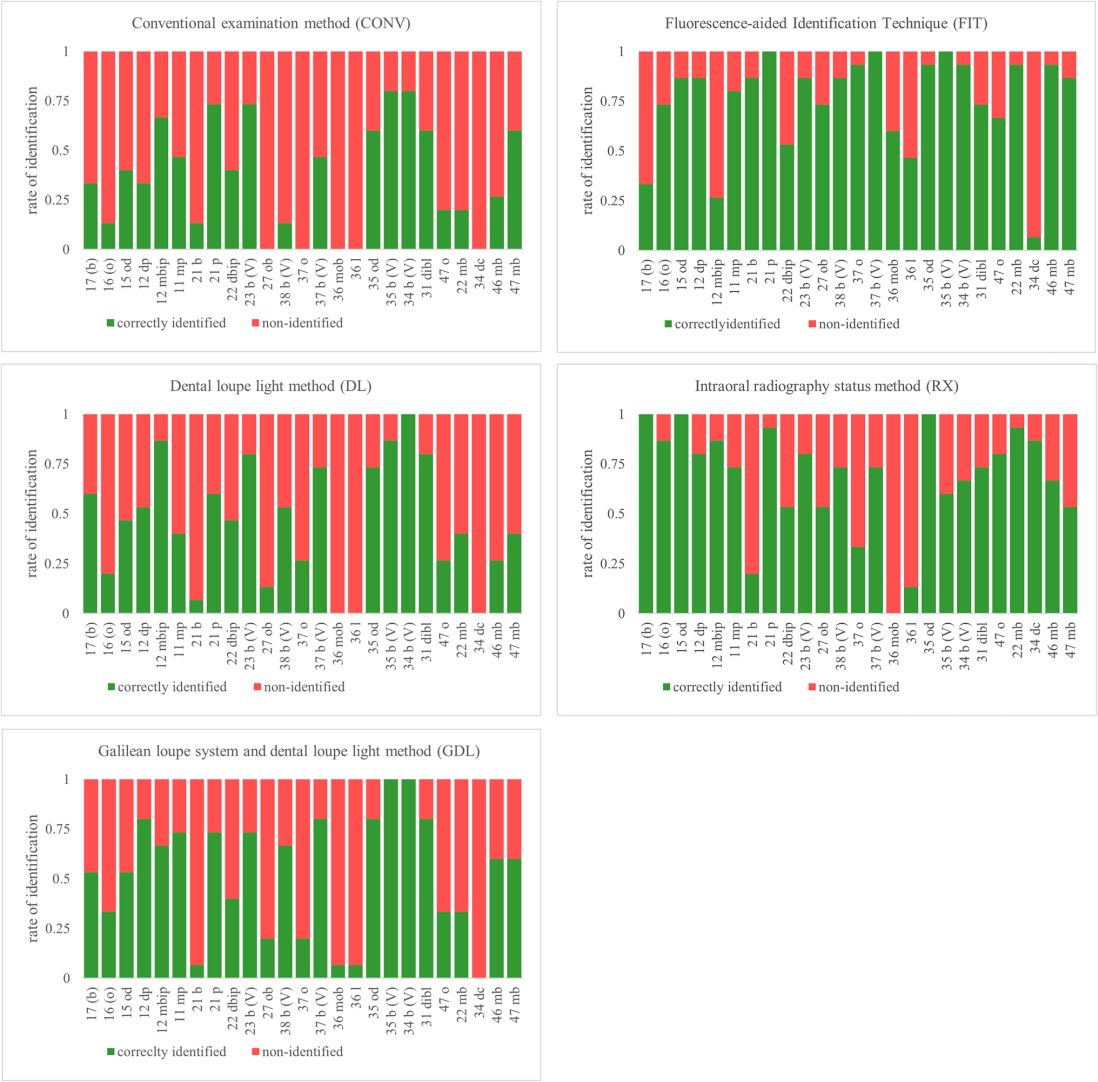
Rate of identification of the composite restorations per method (CONV, DL, GDL, FIT, RX)

**Fig. 4:**
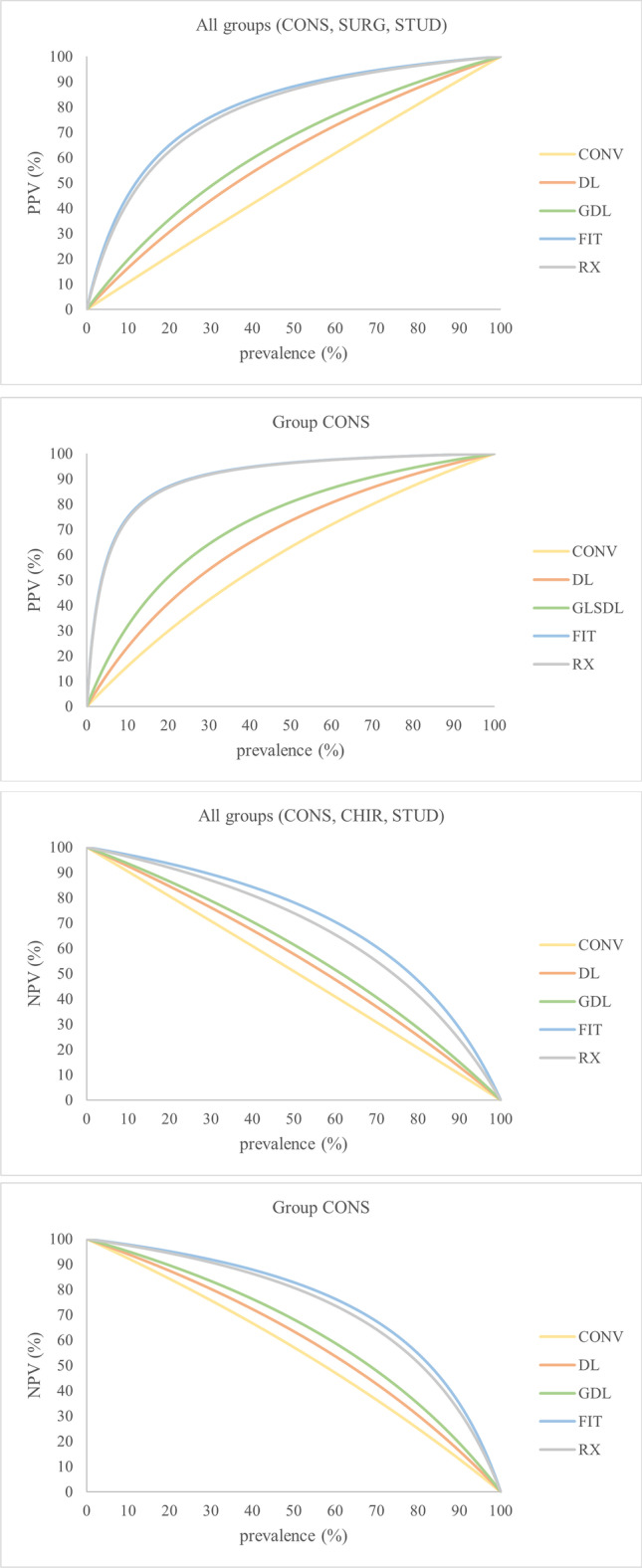
Positive predictive value (PPV) and negative predictive value (NPV) calculated based on prevalence (0–100%)

**Fig. 5:**
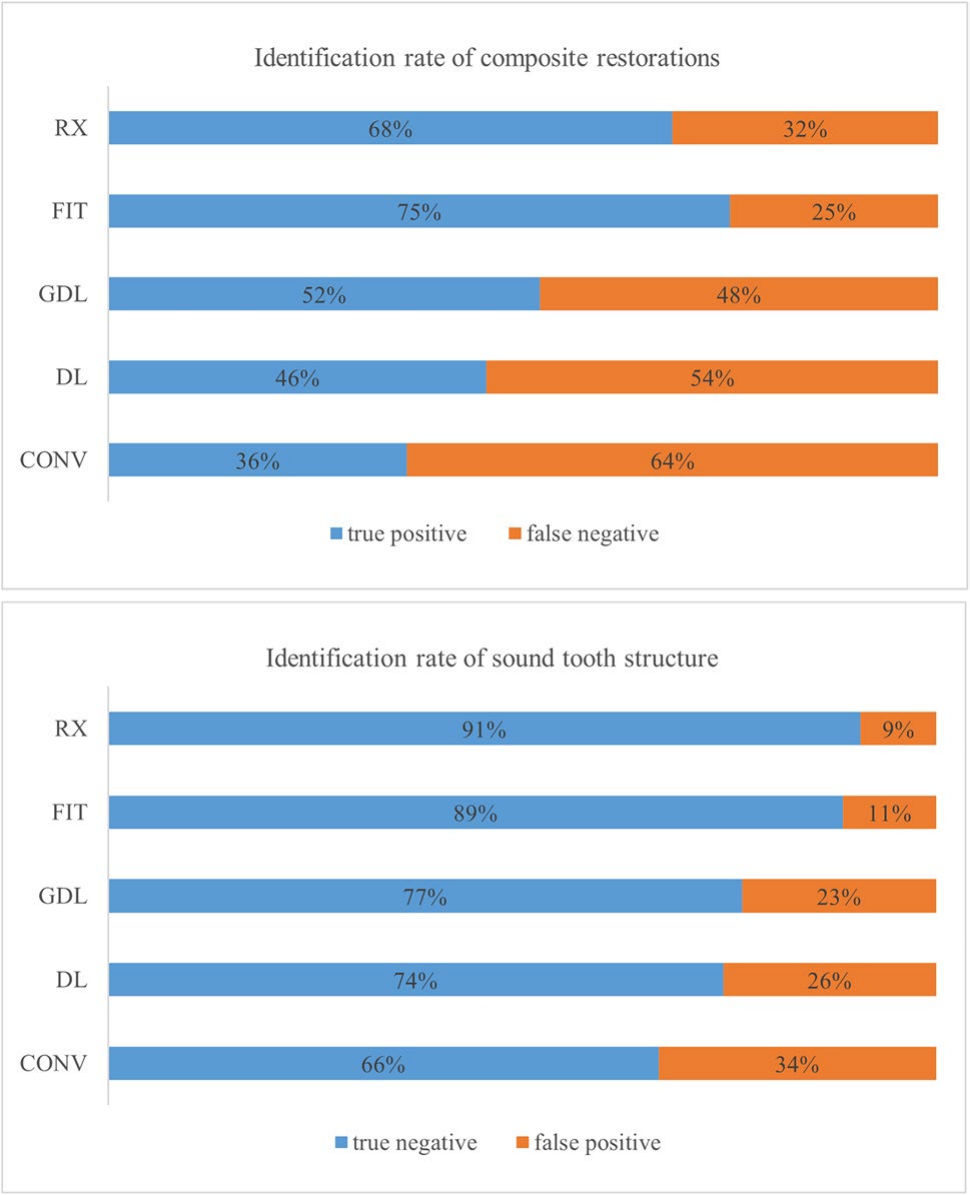
Distribution of true positive and false negative identified composite restorations and true negative and false positive identified sound tooth structures per method

The identification rate (correctly identified versus non-identified) per restoration for each method is presented in Fig. 3. For a comprehensive presentation, the following results are arranged by using the mean values of all groups. Sensitivity, specificity, PPV, and NPV per group and method are shown in Table 4, and *p* values are listed in Table 5. All values were calculated using a filling prevalence of 42%. As the prevalence differs demographically, predictive values for prevalence 0–100% are shown in Fig. 4. Plots for group CONS and the overall values are shown to highlight the impact of the group. The percentual distribution of true-positive and false-negative identified composite restorations and of true-negative and false-positive identified sound tooth structures are given in Fig. 5. The value for the true positive identified composite restorations is identical to the sensitivity, as this parameter describes the accuracy of identifying a composite restoration. Conversely, the specificity describes the accuracy of identifying sound tooth-structure as non-restored and is identical to the true negative value.

**Table 4:** Sensitivity, specificity, PPV, and NPV mean values and values per group

Method	Parameter				
		Group CONS	Group SURG	Group STUD	Mean
CONV	Sensitivity Specificity PPV NPV	45%; CI 37–52% 74%; CI 61–87% 56%; CI 46–67% 65%; CI 61–68%	34%; CI 28–41% 70%; CI 44–97% 52%; CI 22–81% 59%; CI 49–68%	29%; CI 16–41% 55%; CI 12–98% 36%; CI 17–56% 45%; CI 13–78%	36%; CI 31–41% 66%; CI 53–80% 48%; CI 38–58% 56%; CI 47–66%
DL	Sensitivity Specificity PPV NPV	54%; CI 38–70% 81%; CI 63–98% 71%; CI 53–88% 71%; CI 66–76%	46%; CI 29–62% 77%; CI 55–98% 62%; CI 43–81% 66%; CI 56–76%	38%; CI 27–48% 66%; CI 18–114% 55%; CI 25–84% 51%; CI 15–87%	46%; CI 38–53% 74%; CI 61–88% 62%; CI 52–72% 62%; CI 52–73%
GDL	Sensitivity Specificity PPV NPV	60%; CI 40–80% 86%; CI 73–98% 76%; CI 63–90% 75%; CI 67–84%	52%; CI 36–68% 76%; CI 53–99% 65%; CI 40–90% 68%; CI 57–79%	44%; CI 40–58% 68%; CI 18–118% 62%; CI 27–97% 54%; CI 15–93%	52%; CI 44–60% 77%; CI 63–91% 68%; CI 56–79% 66%; CI 55–77%
FIT	Sensitivity Specificity PPV NPV	80%; CI 72–82% 97%; CI 90–103% 95%; CI 86–104% 87%; CI 83–91%	74%; CI 64–85% 91%; CI 83–100% 87%; CI 75–98% 83%; CI 77–90%	71%; CI 48–95% 78%; CI 40–117% 76%; CI 42–111% 76%; CI 47–105%	75%; CI 68–82% 89%; CI 78–99% 86%; CI 76–96% 82%; CI 74–90%
RX	Sensitivity Specificity PPV NPV	77%; CI 61–93% 97%; CI 95–100% 95%; CI 91–99% 86%; CI 77–94%	62%; CI 44–81% 93%; CI 86–99% 85%; CI 70–100% 78%; CI 68–87%	65%; CI 48–82% 83%; CI 64–101% 75%; CI 52–97% 76%; CI 63–89%	68%; CI 60–76% 91%; CI 85–97% 85%; CI 77–93% 80%; CI 75–85%

**Table 5:** *P* values between the methods (CONV, DL, GDL, FIT, RX) based on the mean values

Method	Compared to	Sensitivity	Specificity	PPV	NPV
CONV	DL GDL FIT RX	*p* = 0.149 *p* = 0.011* *p* < 0.001* p < 0.001*	*p* = 0.098 *p* = 0.01* *p* < 0.001* *p* = 0.001*	*p* = 0.018* *p* = 0.004* *p* < 0.001* *p* < 0.001*	*p* = 0.23 *p* = 0.001* *p* < 0.001* *p* < 0.001*
DL	GDL FIT RX	*p* = 0.037* *p* < 0.001* *p* < 0.001*	*p* = 1.000 *p* = 0.002* *p* = 0.041*	*p* = 0.743 *p* < 0.001* *p* < 0.001*	*p* = 0.042* *p* < 0.001* *p* = 0.002*
GDL	FIT RX	*p* < 0.001* *p* = 0.001*	*p* = 0.009* p = 0.104	*p* = 0.001* *p* = 0.001*	*p* < 0.001* *p* = 0.017*
FIT	RX	*p* = 0.147	*p* = 1.000	*p* = 1.000	*p* = 1.000

*Significant difference on significance level α = 0.05

### Sensitivity

CONV showed the lowest sensitivity value (36%; CI 31–41%) followed by DL (46%; CI 38–53%), GDL (52%; CI 44–60%), and RX (68%; CI 60–76%). FIT achieved the highest sensitivity with 75%, CI 68–82%. Significant differences between the methods were shown except for CONV versus DL (*p* = 0.149) and FIT versus RX (*p* = 0.147). A large effect size (η_p_^2^ = 0.792) for the method was found. A medium effect size (η_p_^2^ = 0.062) for the group was shown and a significant difference for CONV between group CONS versus group SURG, *p* = 0.046, and group CONS versus group STUD, *p* = 0.005, both in favor of group CONS.

### Specificity

Specificity values were significantly higher for RX (91%; CI 85–97%) compared to CONV (66%; 53–80%; *p* = 0.001) and DL (74%; CI 61–88%; *p* = 0.041) but not to FIT (89%; CI 78–99%; *p* = 1.000) and GDL (77%; CI 63–91%; p = 0.104). There was no significant difference for CONV versus DL (*p* = 0.098) and DL versus GDL (*p* = 1.000). Effect sizes were small for the groups (η_p_^2^ = 0.028) and large for the method (η_p_^2^ = 0.609) regarding specificity. Group CONS performed significantly better for RX than group STUD, *p* = 0.03.

### Positive predictive value (PPV)

The probability of a detected composite restoration actually being a restoration (PPV) was highest for FIT (86%; CI 76–96%) and significantly higher than for CONV (48%; CI 38–58%; *p* < 0.001), DL (62%; CI 52–72%; p < 0.001), and GDL (68%; CI 56–79%; *p* = 0.001). No significant difference was found between FIT and RX (85%; CI 77–93%; *p* = 1.000) and neither between DL and GDL (*p* = 0.743). A large effect size was found for the method (η_p_^2^ = 0.787) and a medium effect size for the group (η_p_^2^ = 0.054). A significant difference in favor of group CONS between group CONS and group STUD for RX, *p* = 0.024, was shown.

### Negative predictive value (NPV)

Inversely the probability of a sound-tooth being diagnosed as non-restored (NPV) was highest with FIT (82%; CI 74–90%) followed by RX (80%; CI 75–85%) and significantly higher than for CONV (56%; CI 47–66%; *p* < 0.001), DL (62%; CI 52–73%; *p* < 0.001), and GDL (66%, CI 55–77%; *p* < 0.001). The NPV for RX showed no significant difference to FIT.

Large effect sizes were found regarding method (η_p_^2^ = 0.809) and group (η_p_^2^ = 0.194), but no significant differences between the groups.

### Intra- and interrater reliability

The intrarater reliability calculated by using Cohens’ kappa showed for group CONS substantial agreement for CONV (κ = 0.72) and DL (κ = 0.78) and even excellent agreement for GDL (κ = 0.95), FIT (κ = 0.82), and RX (κ = 0.83). Moderate agreement was found in group SURG for CONV (κ = 0.55) and FIT (κ = 0.54) and substantial agreement for DL (κ = 0.68), GDL (κ = 0.63), and RX (κ = 0.64). Group STUD showed moderate agreement for CONV (κ = 0.56), DL (κ = 0.59), and RX (κ = 0.57) and substantial agreement for GDL (κ = 0.61) and FIT (κ = 0.61).

The interrater reliability showed a fair agreement for all methods (κ = 0.267–0.303) calculated by using Fleiss’ kappa.

## Discussion

In order to search for antemortem dental records, a careful postmortem documentation is required. Cases of a complete dentition with only composite restorations have to be identified correctly in order to differentiate sound tooth structure from composite restorations to make identification possible. The similar appearance of teeth and restorations represents a potential error source during forensic examinations. To mistake sound tooth structure for composite restorations (or vice versa) can lead to false records and consequently impede identification, delay investigations, and even lead to wrong identification of a body [1–4, 10, 11]. Therefore, the tooth models in the present study were only reconstructed with composite restorations. This scenario will occur more often in the present and future than in the past. Tooth loss and dental caries are decreasing, and so-called non-restorative or minimal restorative cases are becoming more common [3, 9, 12].

The lowest values for the sensitivity, specificity, PPV, and NPV were shown for CONV. An overall sensitivity of 36% and a specificity of 66% was reached, meaning a third of all composite restorations were correctly identified and a third of non-restored sound tooth structures were misdiagnosed as filled. The group CONS performed significantly better than group SURG and group STUD, regarding the sensitivity, meaning that they correctly diagnosed more of the composite restorations.

For DL where the use of a direct light source was allowed, the sensitivity, specificity, PPV and NPV values increased. A significant improvement was shown for the PPV, indicating that the use of a direct light source enhances the probability of an identified restorations to actually be restored from 48 up to 62%. This method is comparable to the conventional illumination method in the study of Leontiev et al. [24] and Meller et al. [15], who used the same tooth models. Their conventional illumination method contained the use of a dental unit lamp (27′000 Lux). The use of a dental mirror, a sharp explorer, and a multifunctional syringe was, as in this study, permitted. A higher sensitivity of 47% was found by Leontiev et al. than for Meller et al. with 20%. This reflects the results in the present study, where the overall sensitivity for DL was 46%, for the group CONS even higher. The method of illumination matters in case of identifying teeth structures as well as restorations. Meller et al. performed all examinations in a dark room illuminated by artificial light. The ambient light conditions in the present study are comparable to the study of Leontiev et al. In both investigations, the examinations were performed in a room illuminated by the ambient light (500–800 Lux) without direct solar irradiation and in the same building.

As the examinations were performed under daylight and artificial lighting conditions, changes in the ultra-violet components can influence the color of composite resin materials and lead to the fluorescence-induced illuminant metameric failure of composite resin materials. Meaning the composite restoration becomes easier to distinguish from the natural tooth structure [20].

Aged composite restorations may be less difficult to identify even if they were aesthetical perfect in the first place, due to aging of the composite resin material. Aging of the composite restorations in this tooth models cannot be compared to physical aging, although the tooth models were stored in a 0.9% saline solution since their fabrication. Intraoral factors have an additional influence on the in vivo aging.

Good vision seems crucial in dentistry, but there is only small evidence of the improvement of performance by using optical aids [16]. To investigate the benefit of a magnification device for identifying composite restorations, GDL was performed. Galilean loupes with a 2.5 × magnification factor were chosen, as they are the most used optical aid in Swiss dental practice [14, 16, 17, 29]. Sensitivity and NPV values increased significantly compared to DL. Fifty-two percent of all composite restorations were found, and in two-thirds of cases, identified sound tooth structure was actually non-restored. This means that more intact teeth were being diagnosed correctly and a higher accuracy to identify composite restoration occurred. Inferring, using dental loupes could contribute to less false-positive results, which is important for correct identification of a decayed body.

The examiners were familiar with the use of dental loupes and recorded to use them in dental practice, but most with a magnification factor higher than 2.5 × . As the use of dental loupes tends to have a certain adaption phase, the question if the examiners would have reached better results with their own devices remains open. All examiners in the present study were younger than 40 years and had less than 10 years of professional experience in dentistry, so the influence of age and experience cannot be evaluated [29].

As fluorescence-inducing device, the SIROInspect (Dentsply Sirona, York, Pennsylvania, USA) with a spectral bandwidth of 397–411 nm and a peak wavelength of 404 nm was chosen. Such devices are small in size and mobile. Due to their optimal wavelength spectrum (398 ± 5 nm), they are able to detect composite resin materials [21]. There are many affordable devices with a comparable wavelength, which could be easily integrated in daily practice as well as in forensic examinations.

The overall sensitivity value for FIT increased up to 75% meaning that three quarters of all composite restorations were correctly diagnosed. The percentage of false-positive and false-negative results is decreasing, and for all parameters, significant differences to the previous methods were found. The overall sensitivity and PPV values were lower than in the previous investigations, but the values of the group CONS were comparable to the results in the study of Leontiev et al., where dentists working in private practices participated. As conservative dentistry tends to be a main part of general practicing dentists, this could be another indicator of the beneficial influence of experience in this working field [5]. Forensic odontology requires the ability to correctly identify dental restorations, but also to recognize anatomical features, evidence of past trauma, healed fractures or surgical treatment and to assess dental age [4].

Differences between the actual study and Meller et al. [15] and Leontiev et al. [24] may be explained with the aging of the composite resin material, which can lower the fluorescence property of the restoration [30, 31]. But even if the composite resin material does not significantly differ in its fluorescence property, it should still be possible to detect foreign material [12]. Additionally, the fluorescence property is not affected by saliva, blood, or plaque. Therefore, FIT should be considered as a tool in forensic examinations.

Postmortem dental records are made by precise documentation of all dental restorations and structures. If at this point a possible victim is determined, antemortem radiographs are requested before taking postmortem dental radiographs of the deceased. With this procedure it is possible to duplicate the type and angulation of the antemortem radiographs, which is essential for comparison [3, 4, 27]. If postmortem radiographs were already taken for postmortem dental profiling as in this study, retaking the radiographs should be considered for comparison after receiving antemortem radiographs, which can be challenging. The use of x-ray holders is limited as the placement of the holder and its fixation is different. Placement errors and unlike angulations can cause inadequate coverage or overlapping artifacts of the area to be examined radiographically. Both lead to troubles comparing antemortem and postmortem radiographs and should be avoided whenever possible. Radiographs are objective and do not have a subjective influence like a written dental record, but they do not show treatment performed after the last taken image. Any treatment performed should be explainable by treatment demand [3, 4].

Sixty-eight percent of the composite restorations were correctly identified with RX. The group CONS attained even 77%. The specificity reached with an overall value of 91% the highest value compared to the other methods. The probability of an identified composite restoration to actually be restored increased to 85% and for the sound tooth structure to be non-restored to 80%. The group CONS showed significantly higher results than the group STUD regarding the specificity of 97% versus 83% and a PPV of 95% versus 75%. Although there was no significant difference to group SURG, these finding indicate that experience in conservative dentistry tends to improve the ability of recognizing composite restorations in dental radiographs. No significant differences were found between FIT and RX for all parameters.

All composite resin materials used for the tooth models in this study were radiopaque except one layer of the restoration on tooth 12, which seems deniable because of the extension of the restoration. The composite restoration on tooth 34 was excluded in the previous investigations; however, it was considered in the present study, because of the appearance in the radiograph. Two composite restorations made of Amaris (Amaris, VOCO GmbH, Cuxhaven, Germany), displayed a 100% finding rate in RX while none of the 12 restorations made of Tetric Evo Ceram® (Tetric Evo Ceram®, Ivoclar Vivadent AG, Schaan, Liechtenstein) achieved this. Tetric Evo Ceram® shows a higher radiopacity for enamel and dentin, whereas Amaris only for dentin, which would indicate that those restorations would be harder to detect in intraoral radiographs. Especially small composite restorations in enamel or limited to buccal, lingual, or palatal surfaces were less found with RX than with DL, GDL or FIT. This may be due to the effect that the thickness of the restoration influences the observed radiopacity and can still appear similar to enamel or dentin [26]. RX and FIT showed comparable results in identifying composite restorations but depending on their location. The combination of both methods may lead to even better results.

The dentistry students performed significantly lower than the dentists of the group CONS regarding sensitivity values for CONV and specificity and PPV values for RX. There were no differences found between the groups in DL, GDL and FIT. Effect sizes for the group differed from low to high. Yet the dentists of the group CONS showed the highest values for all methods. As the examiner groups in the present study were rather small, clearer results could be accomplished by larger groups.

Even though the same method was repeated in a session, the intrarater reliability showed only excellent agreement in group CONS for GDL, FIT and RX. Other observer groups showed substantial or moderate agreement. The interrater reliability showed an overall fair agreement for all methods.

Postmortem diagnostic can be facilitated by removing the jaws. Especially, to identify small tooth-colored restorations, which are soiled with blood or saliva. Removal of the jaws is recommended for highly destructed bodies but must be compatible with ethical principles [1, 27]. If a jaw resection is not intended, FIT could contribute to identify those small restoration, as they are hard to identify with radiographs. Nowadays lots of dental radiographs are taken digitally, so data transfer is easy and availability is fast. A tool able to search a database of dental panoramic radiographs, compare, filter, and match images has been described [11]. Other authors proposed a semi-automatic method of human identification based on dental radiographs [28]. Unfortunately, this has not been sufficiently studied yet and would require huge databases, which are currently unavailable for a large population. Interpol recommends a software for all, but also dental identification, where semi-automated matches are possible.

These findings highlight the complexity of diagnosing composite restorations and to avoid false documentation of dental records in forensic examinations. Regarding the large effect sizes for all parameters per method, further investigations especially considering the combination of magnification devices, fluorescence-inducing blue-violet light and intraoral radiographs would be necessary to find an optimal set-up to diagnose composite restorations in human teeth.

## Conclusions

Fluorescence-inducing devices show good results in identifying composite restorations and therefore should be considered as a standard tool in forensic examinations.

Intraoral radiographs and fluorescence-inducing blue-violet light showed comparable results in identifying composite restorations but depending on their location. The combination of both methods may lead to even better results.

Good illumination and magnification devices are recommended to enhance performance during conventional examinations.

Involving dentists specialized in conservative dentistry could contribute to dental identification.

### Supplementary Information

Below is the link to the electronic supplementary material.

Supplementary file1 (PDF 217 KB)

## Data Availability

The datasets generated and analyzed during the current study are available from the corresponding author on reasonable request.
