# Supplementary material for: Evaluation of five methods to identify composite restorations in human teeth on a forensic purpose—an ex vivo comparative study
Source: Int J Legal Med. 2022 Aug 10;138(1):85–96. doi: 10.1007/s00414-022-02869-z (PMC10772003; doi:10.1007/s00414-022-02869-z)

Figures

Fig. 1 Mandibular and maxillary tooth model

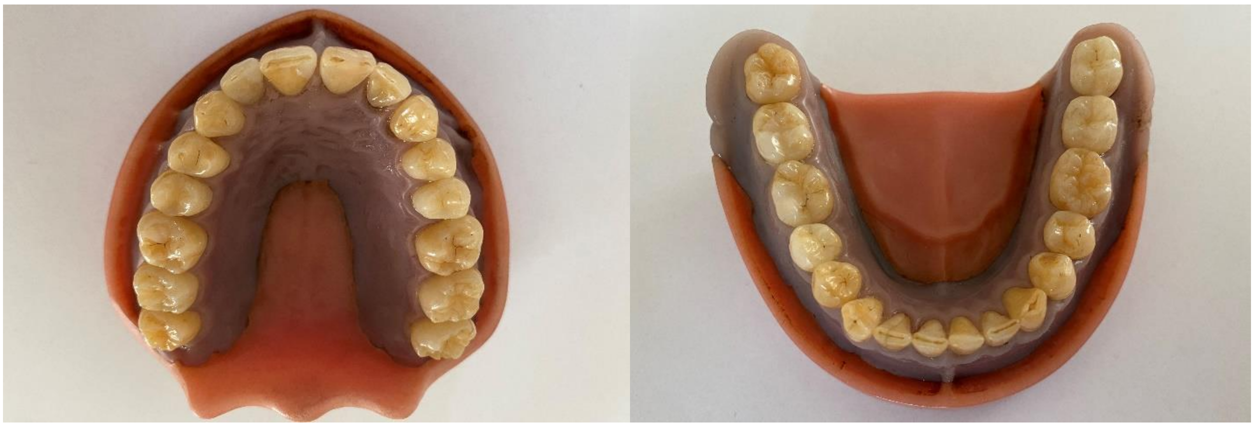

Fig. 2 Intraoral radiography status of the crowns of the tooth model

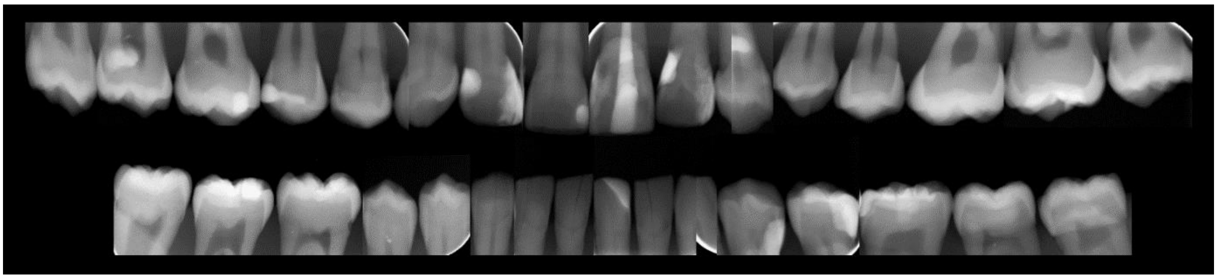

Fig. 3 Rate of identification of the composite restorations per method (CONV, DL, GDL, FIT, RX)

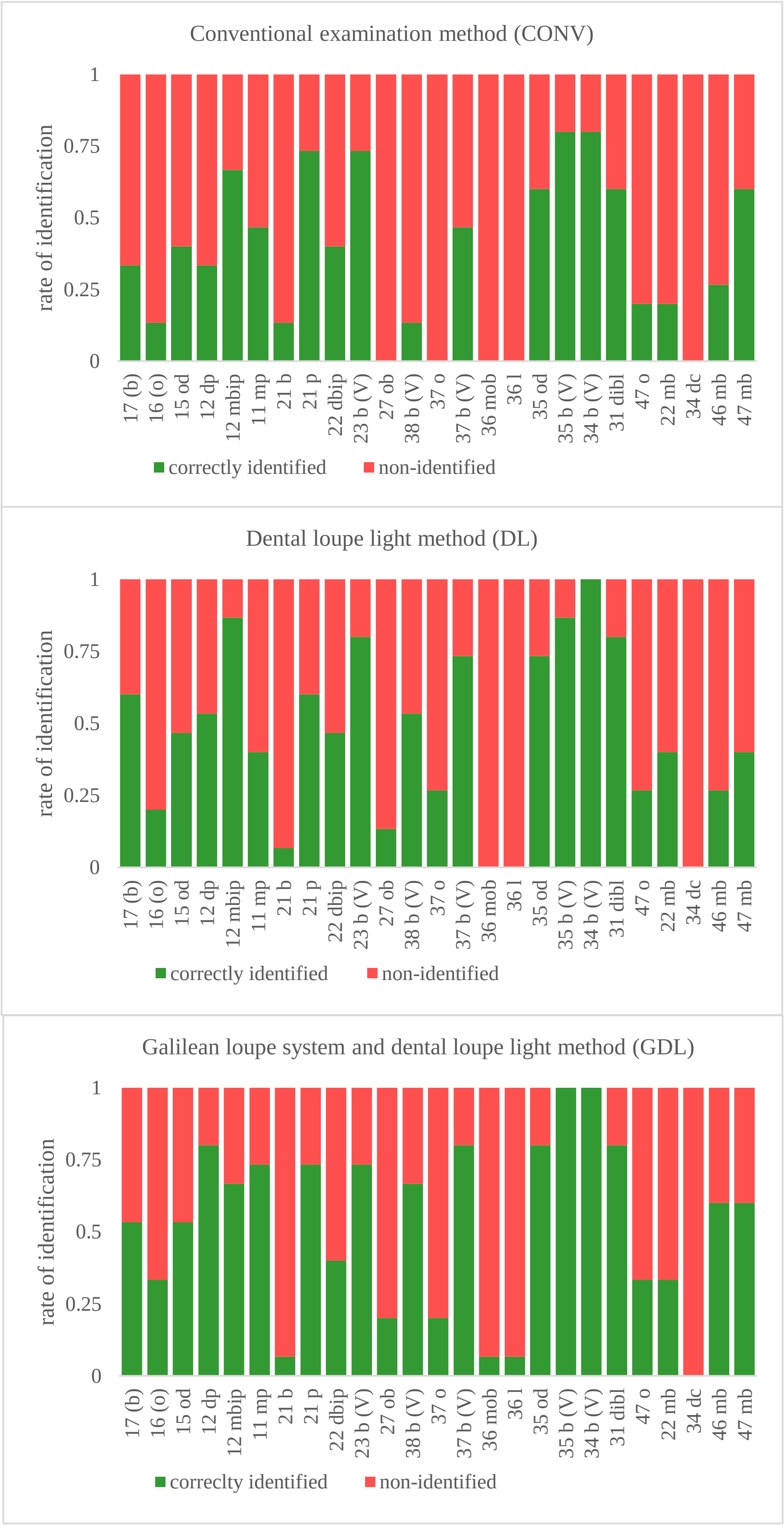

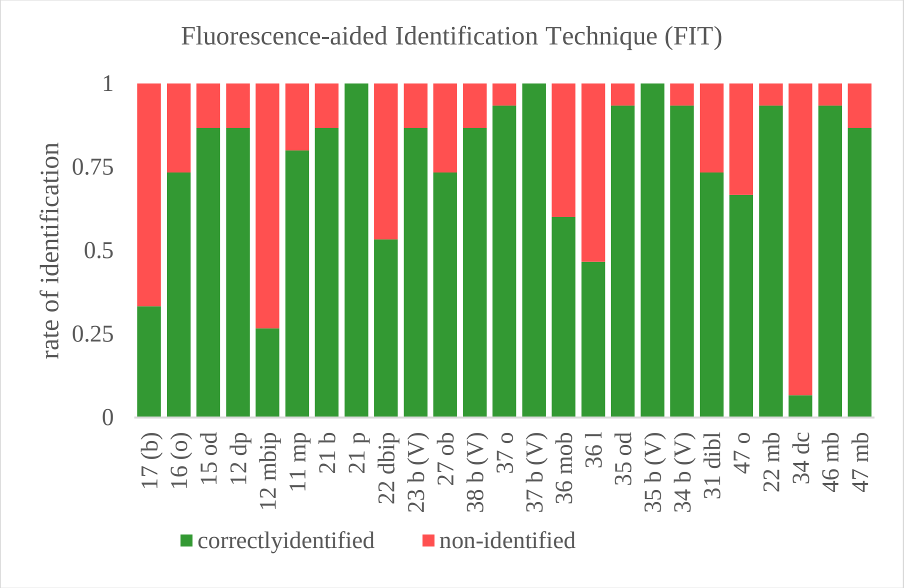

Fluorescence-aided Identification Technique (FIT)
rate of identification
1
0.75
0.5
0.25
0
17 (b)
16 (o)
15 od
12 dp
12 mbip
11 mp
21 b
21 p
22 dbip
23 b (V)
27 ob
38 b (V)
37 o
37 b (V)
36 mob
36 l
35 od
35 b (V)
34 b (V)
31 dibl
47 o
22 mb
34 dc
46 mb
47 mb
correctlyidentified
non-identified

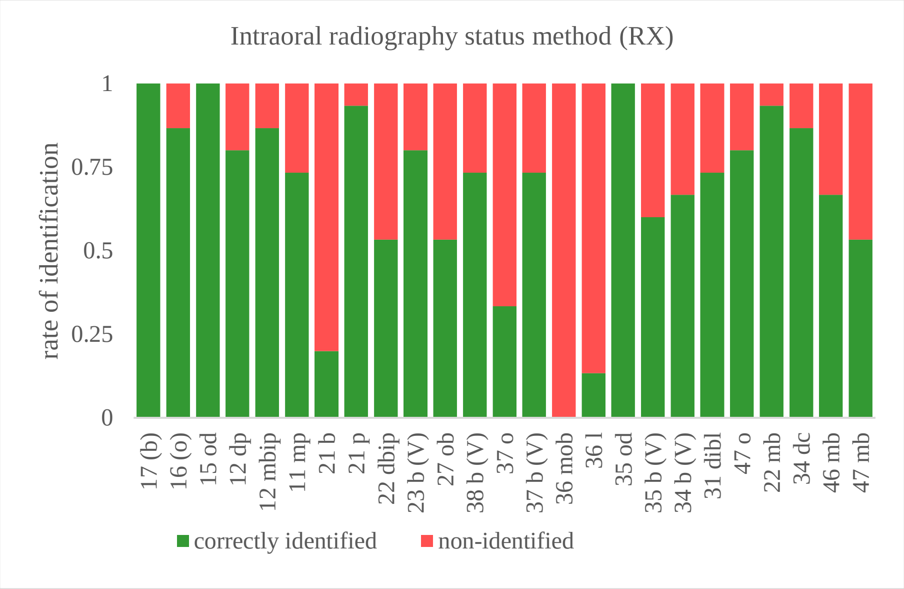

Intraoral radiography status method (RX)
rate of identification
1
0.75
0.5
0.25
0
17 (b)
16 (o)
15 od
12 dp
12 mbip
11 mp
21 b
21 p
22 dbip
23 b (V)
27 ob
38 b (V)
37 o
37 b (V)
36 mob
36 l
35 od
35 b (V)
34 b (V)
31 dibl
47 o
22 mb
34 dc
46 mb
47 mb
correctly identified
non-identified

Fig. 4 Positive predictive value (PPV) and negative predictive value (NPV) calculated based on prevalence (0-100%)

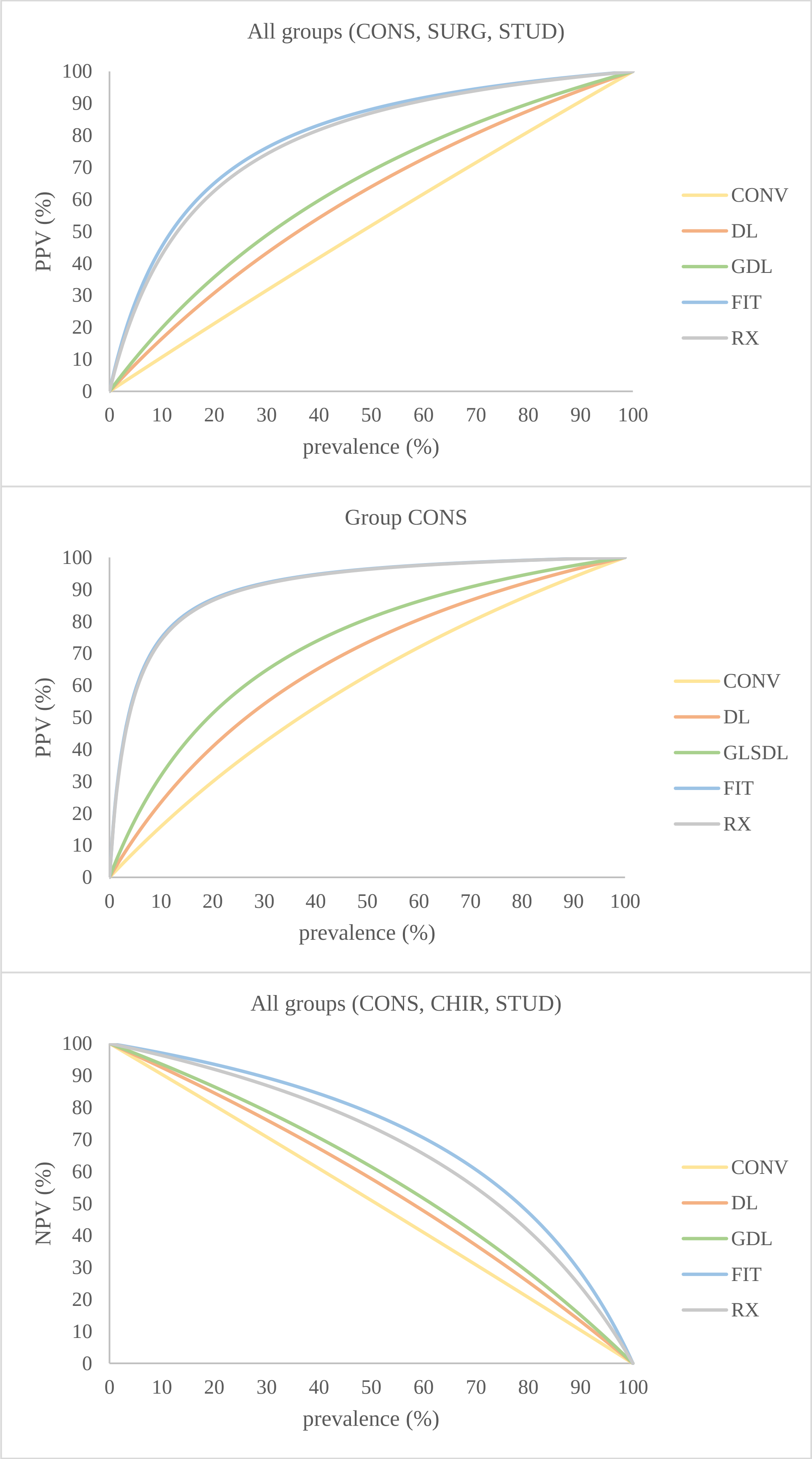

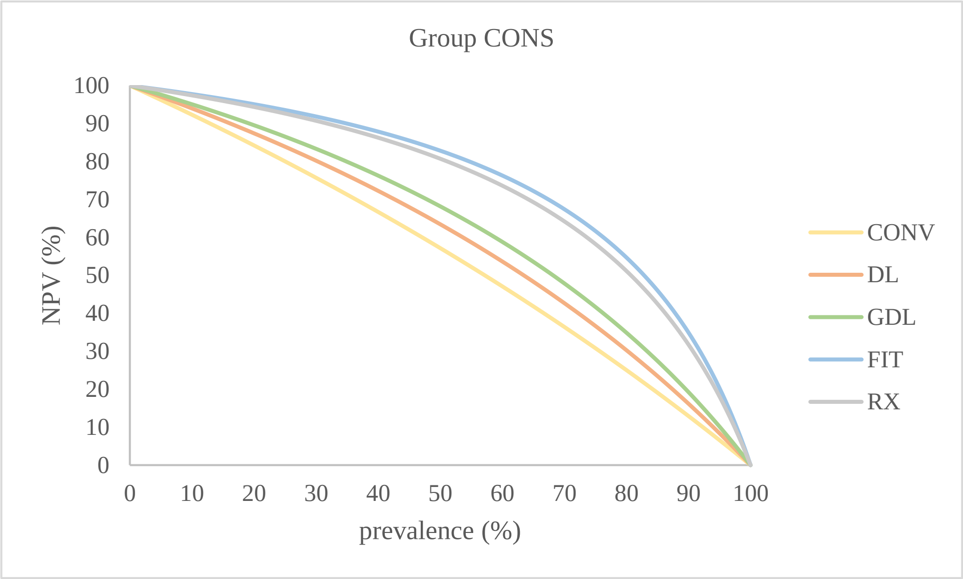

Group CONS
NPV (%)
100
90
80
70
60
50
40
30
20
10
0
0
10
20
30
40
50
60
70
80
90
100
prevalence (%)
CONV
DL
GDL
FIT
RX

Fig. 5 Distribution of true positive and false negative identified composite restorations and true negative and false positive identified sound tooth structures per method

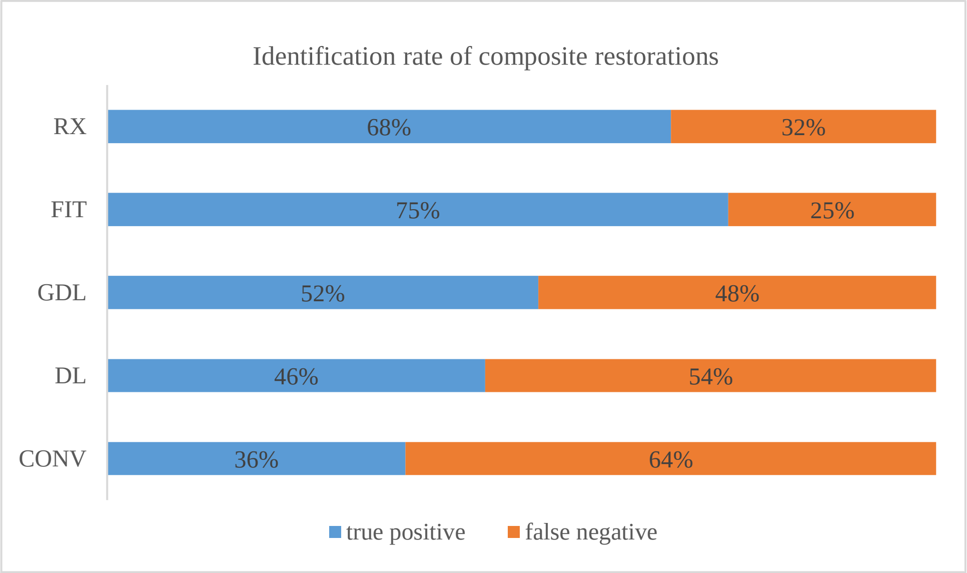

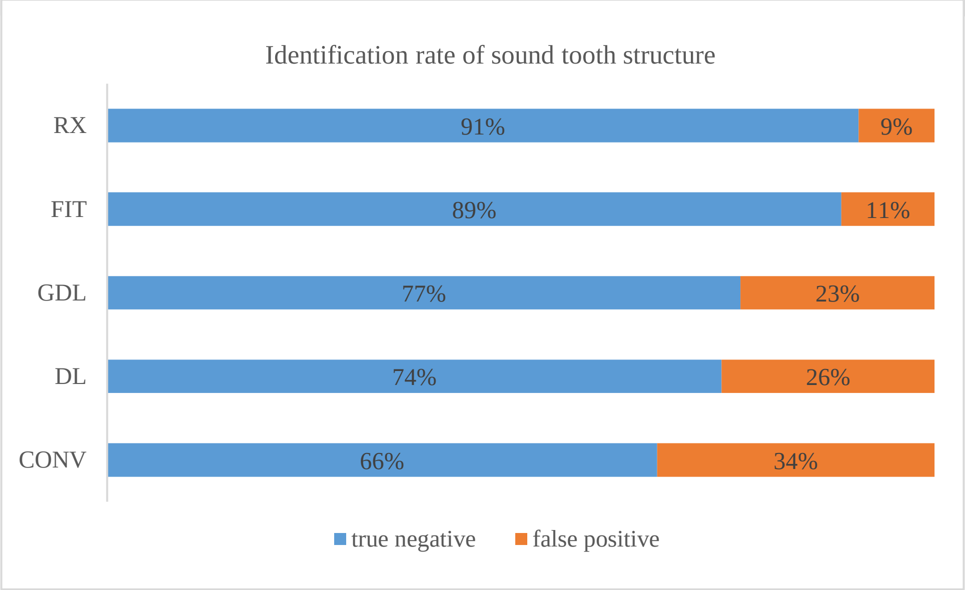

Supplement: Supplementary file 1 — Supplementary file1 (PDF 217 KB) [file 414_2022_2869_MOESM1_ESM.pdf]
